# *In dubio pro silentio* – Even Loud Music Does Not Facilitate Strenuous Ergometer Exercise

**DOI:** 10.3389/fpsyg.2018.00590

**Published:** 2018-05-07

**Authors:** Gunter Kreutz, Jörg Schorer, Dominik Sojke, Judith Neugebauer, Antje Bullack

**Affiliations:** ^1^Department of Music, Carl von Ossietzky University of Oldenburg, Oldenburg, Germany; ^2^Institute of Sport Science, Carl von Ossietzky University of Oldenburg, Oldenburg, Germany

**Keywords:** music listening, ergometer, loudness, perceived effort, hearing prevention

## Abstract

**Background:** Music listening is wide-spread in amateur sports. Ergometer exercise is one such activity which is often performed with loud music.

**Aim and Hypotheses:** We investigated the effects of electronic music at different intensity levels on ergometer performance (physical performance, force on the pedal, pedaling frequency), perceived fatigue and heart rate in healthy adults. We assumed that higher sound intensity levels are associated with greater ergometer performance and less perceived effort, particularly for untrained individuals.

**Methods:** Groups of high trained and low trained healthy males (*N* = 40; age = 25.25 years; *SD* = 3.89 years) were tested individually on an ergometer while electronic dance music was played at 0, 65, 75, and 85 dB. Participants assessed their music experience during the experiment.

**Results:** Majorities of participants rated the music as not too loud (65%), motivating (77.50%), appropriate for this sports exercise (90%), and having the right tempo (67.50%). Participants noticed changes in the acoustical environment with increasing intensity levels, but no further effects on any of the physical or other subjective measures were found for neither of the groups. Therefore, the main hypothesis must be rejected.

**Discussion:** These findings suggest that high loudness levels do not positively influence ergometer performance. The high acceptance of loud music and perceived appropriateness could be based on erroneous beliefs or stereotypes. Reasons for the widespread use of loud music in fitness sports needs further investigation. Reducing loudness during fitness exercise may not compromise physical performance or perceived effort.

## Introduction

Music listening during every-day activities is a global phenomenon in present-day leisure and sports cultures (Kurmaeva, 2011). Background music appears to play an ambiguous role as a distractor that can interfere with cognitive tasks (e.g., Cho, 2015) or enhance physical performance (e.g., Copeland and Franks, 1991). Therefore, the overall effectiveness of background music in mediating psychological processes has been questioned (Behne, 1999), pointing to the importance of psychological attributions such as liking or preference (e.g., Stratton and Zalanowski, 1984; Kreutz et al., 2007; Zatorre and Salimpoor, 2013) and prior exposure (Crust, 2004) on the one hand, and musical features such as tempo, sound intensity, and loudness (e.g., Copeland and Franks, 1991; Waterhouse et al., 2010; Thompson et al., 2012; Metcalfe, 2016) on the other. Here, we investigate some of these issues in the context of physical ergometer exercise, in which participants were exposed to background music of varying sound intensity levels.

Audio-based interventions have become a much debated topic in sport science approaches to enhance performance in a variety of domains (Sors et al., 2015). Specifically, auditory action–perception coupling as part of more general research on the role of natural movement sounds in sports has been studied across various sports domains including basketball (Camponogara et al., 2017), fencing (Allerdissen et al., 2017), elite rowing (Schaffert et al., 2011), ball sports (Sors et al., 2017, 2018), and tennis (Cañal-Bruland et al., 2018). For example, auditory information can improve fencers’ prediction of attack movements (Allerdissen et al., 2017), enhance the performance in hammer throwing (Agostini et al., 2004), or facilitate long-term storage of individual movement patterns in hurdling (Pizzera et al., 2017).

Researchers have pointed out the importance of self-generated movement sounds in action–perception coupling in the sense that such sound cues can help to discriminate between one’s own movements and movements from other sources (Murgia et al., 2012; Kennel et al., 2014a,b). Note that loudness is one auditory attribute that seems of particular relevance in sports as greater loudness can improve reaction times (Brown et al., 2008), or influence referees’ judgments in team sport games (Unkelbach and Memmert, 2010).

Kämpfe et al. (2011) conducted a review and meta-analysis of the psychological and behavioral effects of background music across a wide range of cognitive and physical tasks. Generally, the hypothesis of a modulating effect of background music was not confirmed. However, background music in sports was one of few domains showing a small but positive impact on performance. By contrast, Brooks and Kristal (2010) concluded from their review of studies on music listening exclusively in the field of sports that the evidence of motivational effects of music listening in sports was mixed. This means that music can also be perceived as disturbing or interfering with sports activities. Therefore, the perceived appropriateness and objective effectiveness of music listening in sports activities can be modulated by a range of variables. Moreover, the individual level of training status can also influence the psychological effects of music listening during sports exercises (Baldari et al., 2010).

Studies showing that music listening may have motivating effects to enhance physical performance and reduce perceived effort have focused on individual sports (Karageorghis et al., 2006; Terry et al., 2012). For example, Karageorghis et al. (2008) found that music listening in running could be motivating, but self-selection, preference, and tempo were important moderating variables. Waterhouse et al. (2010) showed that manipulating the musical tempo during ergometer cycling also modulated performance in the sense that increasing the tempo led to greater distances covered and more positive affective experience. Barwood et al. (2009) found that music could distract gym users from bodily perceptions and provide motivation to enhance performance during motorized treadmill exercise. These authors showed that runners covered significantly more distance in a motivational music (and video) condition as compared to non-motivational and control conditions.

Most study designs entail participant’s exposure to recorded or live music. Fritz et al. (2013), however, tested a novel music agency concept, in which fitness devices were equipped with sound processing software such that movement of the devices during exercise controlled the production of synthesized sound and thus provides a music feedback. The authors compared psychophysiological responses to the music listening with feedback versus music listening without such feedback. They observed that the ratio of performance and subjective exertion was significantly more favorable in the feedback condition and concluded that music agency was an efficient strategy to enhance pleasantness of strenuous exercise (Fritz et al., 2013).

Listening to background music in sports and fitness contexts is not without risk. There is controversial debate as to whether induced hearing loss may be attributable to music listening for leisure purposes (Zhao et al., 2010; Carter et al., 2014). Some authors maintain that prolonged exposure to high sound pressure levels might pose a threat to hearing especially for younger people (Vogel et al., 2007; Petrescu, 2008). Specifically, fitness instructors were found particularly prone to attract hearing problems through their profession (Nie and Beach, 2016). Consequently, attendance at fitness studios has been explicitly included in a portfolio of potentially harmful activities for adolescents’ and young adults’ hearing (Beach et al., 2013).

The motivations for listening to loud music and the preference for higher as opposed to lower volume levels are unclear. Todd and Cody (2000) found that high volume levels of dance music were associated with vestibular responses to low-frequency beats. They assume that such responses could reach the pleasure centers of the brain via the thalamus. Studies of the behavioral characteristics of loud music consumers reveal indications of addiction in a proportion of excessive listeners (Florentine et al., 1998). The marginal evidence supporting favorable psychological effects of loud music notwithstanding, production and dissemination strategies in the music and broadcasting industries seem to adhere to the notion that music listeners under most circumstances might prefer louder over the softer music of the same kind (Vickers, 2010; Katz, 2015).

Metcalfe (2016) undertook one of the few studies to investigate the influences of different intensity levels (45 and 75 dB) on walking speed but found no systematic influence of this variable. However, this study did not include a silent condition and participants’ subjective levels level of exertion were not assessed. In another study comparing the differential effects of loud vs. soft music on subjective experience during a treadmill exercise, Edworthy and Waring (2006) observed that music *per se* had a significant impact on positive affect, but not on perceived exertion. However, based on their findings, these authors recommend loud music to optimize the affective experience of work-out in the gym.

The present study used a broader range of intensity levels, included participants with varying sports experience, and also entailed measures of physical performance and perceived effort during a rigorous ergometer exercise. Therefore, despite the negative findings by Metcalfe (2016), increases in performance and decreases in the perceived effort were expected to be associated with higher intensity levels as compared to lower levels. We also took measures to ascertain the appropriateness of the music from the participant’s point of view. Finally, a physiological measure (heart rate) during task performance was used as a proxy for the participants’ fitness levels.

### Aim, Research Questions, and Hypotheses

The central aim of the study was to investigate the influences of electronic dance music of different loudness levels on physical, behavioral, and physiological responses in trained and untrained healthy adults during ergometer exercise. Hence, we ask to what extent loudness modulated an aerobic ergometer performance. We further were interested in how the presence of music *per se* was perceived as appropriate in terms of loudness and tempo, preferable, and motivating during the exercise for two groups of different skill levels. Despite the mixed evidence in favor of positive effects of music listening during sports exercise, we nevertheless assumed that louder music leads to (a) significantly greater output and (b) significantly reduced perceived effort as compared to both exposure to softer music or no music.

## Materials and Methods

### Participants

Forty males at the age between 19 and 35 years (*M* = 25.25 years; *SD* = 3.89 years) were recruited from the University of Oldenburg. These participants were classified into two different groups. Group 1 consisted of male handball players that played on a medium level of skill and can be categorized as advanced players with at least 4 h of training per week. For the second group, students were recruited that did no sports on a regular basis. All participants reported normal hearing conditions, no cardio-vascular diseases, impairments of the locomotor system, or intake of mind-altering medication. Before testing, every individual participant provided written consent to participate in the study.

### Stimulus Material

A selection of three music pieces was used in this study: (1) Roxfield “Freak Out” (stone mix), (2) Robbie Moroder featuring Anna Carels “Fucking hands up,” and (3) Paranoja Crank House Stage “Infinity.” The selection was strategic as representing modern electronic dance music that is typical for functional use in sports contexts. As expected, the pieces were unfamiliar to the majority of participants. The tempi and dynamics of these songs were adjusted to 128 beats per minute with a standard software (Audacity and logic pro X). The stimuli were presented by an Apple “MacBook Pro Notebook” via dB Technologies© “Twin 128” stereo-loudspeakers. Sound emission was measured in the vicinity of participant’s head by using a Testo© “816-1” sound pressure level meter. Sound intensity was adjusted such that it represented the average intended dB-level in the music conditions.

### Measurement Instruments

#### Equipment

The Cyclus 2^®^ ergometer was used to evaluate physical performance in Watt (W), the force on the pedal in Newton meter (Nm), and the pedaling frequency as revolutions of the crank per minute (rpm). The ergometer was combined with a frame of a Felt^®^ racing cycle (size 56) equipped with a Shimano^®^ ‘Sora’ gear change. Data were read out from the ergometer via a USB-port and transferred to a desktop computer.

The Polar^®^ ‘RS400’ heart rate monitor watch in connection with a chest belt ‘Wearlink 31’ was used to examine heart rate measured in beats per minute (bpm). Data were transferred to a computer via a USB-port and analyzed using the ‘ProTrainer5’ software package provided by Polar^®^. Subsequently, the data were exported and combined with the ergometer data file.

#### Questionnaires

A brief questionnaire was developed to collect information about the age of the participants and the regularly performed sportive activities. The health status was ascertained with the ‘Health check questionnaire,’ developed by the German Society for Sports Medicine and Prevention [Deutsche Gesellschaft für Sportmedizin und Prävention (DGSP)]. During the experiments, participants rated their current perception of fatigue and acoustical environment. Fatigue (“How exhausted are you in this moment?”) was evaluated on a nine-point likert scale with 1 equalling “I feel really exhausted now” to 9 “I do not feel exhausted now at all.” The second question concerned their perception of the acoustical environment (“How pleasant do you perceive your acoustical environment?”) was rated again on a nine-point likert scale, with 1 equalling ‘really unpleasant’ and 9 being ‘really pleasant.’ Furthermore, after the experiment, participants gave information about the perceived loudness (“Was it too loud during the experiment?”) and the familiarity with the music pieces (“Were you familiar with one of the presented musical pieces during the experiment?”). In addition, they indicated their subjective perception during the experiment based on three questions rated on five-point likert scales. The motivating effect of the music (“I found the music…”) was rated on a scale labeled with ‘disturbing’ (1), ‘rather disturbing’ (2), ‘irrelevant’ (3), ‘rather motivating’ (4), and ‘motivating’ (5). Categories for the appropriateness of the genre (“The music genre was for this sport…”) were labeled with ‘inappropriate’(1), ‘rather inappropriate’ (2), ‘suitable’ (3), ‘rather appropriate’ (4), and ‘appropriate’ (5). Ratings of the general impact and the perception of the music genre averaged with higher values representing greater impact of the respective measure. Further, the tempo was assessed on a scale labeled ‘too slow’ (1), ‘slow’ (2), ‘appropriate’ (3), ‘fast’ (4), and ‘too fast’ (5). These ratings were also averaged. A response in the middle of the scale indicates an ideal tempo.

### Procedure

**FIGURE 1:**
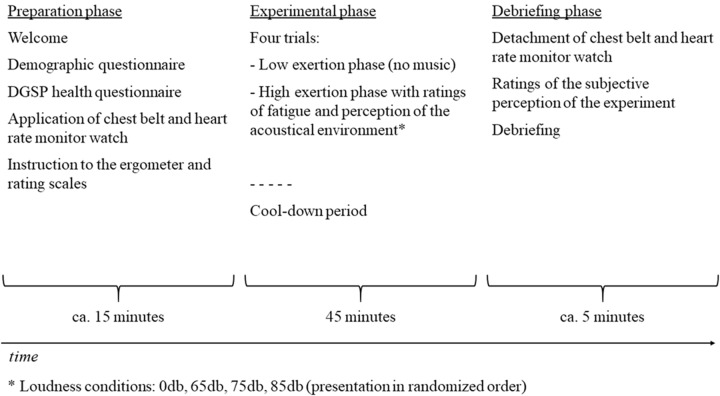
Time line of experimental sessions.

Participants were tested in single sessions in the sports science lab of the University of Oldenburg. Upon arrival, they gave informed consent and filled the demographic questionnaire as well as the DGSP health questionnaire. The latter was immediately evaluated by a research assistant to ensure an uncritical participation. Subsequently, participants changed their clothes and were instructed how to apply the chest belt. The heart rate monitor watch was applied to the left wrist. Before the experiment started, participants were inducted into to the ergometer and the rating scales. After the research assistant calculated the individual maximal pulse, the task started with the low exertion phase as a warm-up. To define the two varying physical loads in the experimental phases, the maximal pulse was calculated by using a formula developed by Spanaus (2002). The maximal pulse equals: 214 – (0.5 × [age of participant in years] – 0.11 × body weight [in kilograms]). Hence, low exertion was represented by 60–65% of the maximal pulse, whereas and high exertion was represented by 80–85% of the maximal pulse. In the low exertion phase, no music was played. In the high exertion phase, the physical load was increased to the target pulse range. If the participants exceeded this range, the resistance in the ergometer was adjusted accordingly. The high exertion phase consisted of four different loudness conditions. In condition 1, music was still at 0 dB; in condition 2, a first song was played at an average sound pressure level of 65 dB. In conditions 3 and 4, the sound intensity was increased in 10-dB-steps to 75 and 85 dB intensity, respectively. Each intensity level was marked also with a new song. The order of the four conditions, as well as the order of the songs, was randomized for all participants. The two phases alternated four times. Each phase lasted 5 min. Thirty seconds before the phases ended the experimenter asked the participant to rate their current perception of fatigue and to evaluate the acoustic environment. After the last low exertion phase, participants filled the questionnaire about their subjective perception during the experiment. Every participant was provided with a cash incentive of 8.00 €. Each session lasted about 65 min in total. **Figure 1** depicts the time line of the experimental session.

This study was carried out in accordance with the recommendations of the Carl von Ossietzky University’s Ethics Committee. This committee approved the protocol of the current study. All subjects gave written informed consent in accordance with the Declaration of Helsinki.

### Data Preparation and Analysis

Physical performance, force on the pedal, and pedaling frequency data were exported from the ergometer as CSV-data files. Mean values for every condition were calculated. When heart rates fell out of the target range of 80 to 85% during the high exertion condition for more than a third of the training session, physical measures were excluded from further analysis. First, a one-way ANOVA with repeated measures was conducted across all dependent variables to detect effects of order. Dependent variables were analyzed by a 4 × 2 repeated measures ANOVA with group (low and high trained) as a between-subjects and condition (0 dB/65 dB/75 dB/85 dB) as within-subject factor. Preconditions for conducting ANOVAs were assessed (normality Box’s M test of equality of covariance matrices and Mauchly’s test of sphericity). Accordingly, degrees of freedom were estimated in the *F*-statistics using Greenhouse–Geisser corrections where appropriate. Bonferroni’s test was used for *post hoc* comparisons of means. In all statistical tests, *p*-values were set to 0.05. In addition, partial eta-square was calculated as a measure of the effect size.

G^∗^Power (Faul et al., 2009) was used to conduct an *a priori* power analysis using the *F*-tests function and the algorithm for ANOVA (repeated measures, within-between interactions). According to this program, a total samples size of 36 participants was needed to obtain an effect size of f = 0.25 [α-level: 0.05, Power (1 – β): 0.95, correlations among repeated measures: 0.5]. Due to differences from targeted heart rates one participant had to be excluded from the 65 dB condition and two participants from the 75 dB condition.

## Results

**Table 1** summarizes the descriptive statistics for physical and psychological measures across low and high trained groups during the four exertion phases. No effects of sound intensity on physical performance and force on the pedal were found, all *F*s < 0.68, all *p*s > 0.54. However, there was a trend for the main effect in terms of pedaling frequency, *F*(2.45,86.14) = 2.65, *p* = 0.07, $\eta_{p}^{2}$ = 0.07. Bonferroni-adjusted *post hoc* tests indicated significantly lower frequencies during the 0 dB condition [CI 95% (66.18, 75.33)] in comparison to the 75 dB condition [CI 95% (69.29, 78.90)].

**Table 1:** Means (and standard deviations) of physical and psychological measures across low and high trained groups during different conditions.

	0 dB	65 dB	75 dB	85 dB
Physical performance				
Low trained	150.89 (37.93)	145.65 (32.71)	149.77 (33.22)	145.30 (35.42)
High trained	202.16 (45.26)	200.00 (41.39)	196.24 (40.36)	195.15 (46.17)
Overall	178.60 (48.90)	175.03 (46.19)	174.89 (43.61)	172.25 (48.14)
Force on the pedal				
Low trained	127.48 (45.68)	123.65 (32.65)	122.03 (39.84)	122.42 (49.82)
High trained	159.36 (45.91)	162.85 (49.23)	146.74 (42.86)	155.24 (62.07)
Overall	144.71 (47.95)	144.84 (46.32)	135.39 (42.79)	140.16 (58.40)
Pedaling frequency				
Low trained	68.78 (14.03)	67.58 (14.55)	71.43 (14.68)	71.99 (20.35)
High trained	72.72 (13.36)	71.22 (14.53)	76.76 (14.06)	74.38 (15.78)
Overall	70.91 (13.62)	69.55 (14.45)	74.31 (14.40)	73.28 (17.81)
Fatigue				
Low trained	4.45 (1.93)	4.50 (2.04)	5.00 (1.65)	4.40 (1.93)
High trained	4.75 (1.86)	4.95 (1.61)	5.00 (1.69)	4.95 (1.85)
Overall	4.60 (1.88)	4.73 (1.83)	5.00 (1.65)	4.68 (1.89)
Acoustical environment				
Low trained	4.40 (2.26)	5.45 (1.61)	6.10 (2.10)	5.70 (2.36)
High trained	4.30 (2.06)	5.90 (1.97)	6.40 (1.90)	6.55 (2.09)
Overall	4.35 (2.13)	5.68 (1.79)	6.25 (1.98)	6.13 (2.24)


*Physical performance are measured in Watt (W), the force on the pedal in Newton meter (Nm), and the pedaling frequency as revolutions of the crank per minute (rpm).*

### Order and Time Effects

There were significant effects of order regarding physical performance, *F*(1.86,66.77) = 60.05, *p* < 0.001, $\eta_{p}^{2}$ = 0.63, force on the pedal, *F*(1.40,50.55) = 32.50, *p* < 0.001, $\eta_{p}^{2}$ = 0.47, and pedaling frequency, *F*(1.68,60.47) = 8.70, *p* < 0.001, $\eta_{p}^{2}$ = 0.20. While physical performance and force on the pedal significantly decreased over time, an increase of pedaling frequency was observed. In addition, perceived fatigue increased over time, *F*(2.47,96.26) = 28.99, *p* < 0.001, $\eta_{p}^{2}$ = 0.43.

### Training Level and Music Stimulation

Two-factorial ANOVA including training level and presence or absence of music during ergometer exercise were calculated for each of the dependent measures. To these ends, the three music conditions (65, 75, and 85 dB) were averaged and mean values entered into the analyses. There were significant main effects for physical performance, *F*(1,76) = 35.41, *p* < 0.001, and force on the pedal, *F*(1,76) = 12.40, *p* < 0.001. No further main or interaction effects were observed for the remaining dependent variables.

### Perception of the Acoustic Environment

There was a significant main effect for perceived appropriateness of the acoustic environment, *F*(2.15,81.70) = 8.68, *p* < 0.001, $\eta_{p}^{2}$ = 0.19. Bonferroni-adjusted *post hoc* tests revealed significant differences between the 0 dB condition [CI 95% (3.66, 5.04)] and all other conditions, namely 65 dB [CI 95% (5.10, 6.25)], 75 dB [CI 95% (5.61, 6.89)], and 85 dB [CI 95% (5.41, 6.84)]. Results showed lowest ratings in the 0 dB conditions and highest ratings in the 75 dB condition (see **Table 1** for details).

### Music Evaluations

Majorities of participants rated the music as not too loud (65%) and unfamiliar (97.50%). The music was rated as quite motivating (*M* = 3.85, *SD* = 1.00) and appropriate for this sports exercise (*M* = 4.00, *SD* = 0.99). The tempo of 128 bpm was perceived as appropriate (*M* = 3.25, *SD* = 0.59). **Table 2** summarizes the descriptive statistics for subjective responses. No differences between groups occurred, all *ts* < 0.01 and *ps* > 0.20, except for the perception of tempo, *t*(36.96) = -2.26, *p* < 0.05, *d* = 0.72. High trained participants (*M* = 3.45, *SD* = 0.61) perceived the music significantly faster as low trained participants (*M* = 3.05, *SD* = 0.51). However, ratings of both groups are in a positive range.

**Table 2:** Means (and standard deviations) of subjective ratings across low and high trained groups.

	Rating scales		
	Motivation	Appropriate for sports exercise	Appropriate tempo of music
Low trained	3.85 (0.99)	4.00 (1.12)	3.05 (0.51)
High trained	3.85 (1.04)	4.00 (0.86)	3.45 (0.61)
Overall	3.85 (1.00)	4.00 (0.99)	3.24 (0.59)


*Scales range from 1 to 5. Higher values of motivation and genre appropriateness representing greater impact of the respective measure. Concerning tempo, a response in the middle of the scale indicates an ideal rating.*

## Discussion

We asked whether music listening facilitated the performance and experience of strenuous ergometer exercise in trained and untrained healthy males. We assumed that music listening induced positive effects with respect to physical and subjective measures in the sense that loud music enhances performance and reduces perceived stress or effort. It was ensured that the music selection for this trial was appropriate and acceptable to the participants. And we took measures that the exercise was strenuous thus reflecting a typical workout protocol. Despite these efforts to construct a laboratory trial with high ecological validity, we failed to find unequivocal patterns of positive effects of music listening during the trial and across participant groups.

The observation that presenting music at 0 or 85 dB did not lead to any significant differences in the dependent measures of this study has important theoretical and practical implications that may warrant both further investigation and reconsideration of the use of music during fitness exercise. Theoretically, music listening may still have positive effects, but the mechanisms causing such effects are yet unclear. Practically, policies of the use of music in fitness studios particularly with respect to their intensity levels and potential risks for exercisers should be reconsidered. We will discuss these points in turn.

First of all, it is of note that our findings are in conflict with previous work which suggests more beneficial effects of loud music on performance during sports exercise (e.g., Edworthy and Waring, 2006). The rationale of such observations and interpretations is that loudness enhances the arousal potential of music stimulation and facilitates to distract attention from bodily perceptions to external cues (e.g., Murgia and Galmonte, 2015). However, previous evidence suggesting that loud music might reduce perceived exertion, or enhance physical aspects of performance, appears rather limited. To our knowledge, the current study is one of the first to systematically address this issue. Our results suggest that the hypothesis of performance enhancing effects of loud music during strenuous ergometer exercise must be rejected.

Fritz et al. (2013) have argued that music listening cannot be understood as a mere distraction, but instead can evoke brain mechanisms that lead to releases of hormones to reduce the perception of strain and enhance the experience of positive emotions. This interpretation is grounded on a body of research which has shown that music that is perceived as highly pleasurable can evoke brain systems associated with reward and emotion (e.g., Blood and Zatorre, 2001; Zatorre and Salimpoor, 2013). These observations resonate with potentially pain-reducing effects of more active music behaviors such as singing (Weinstein et al., 2016) or dancing (Tarr et al., 2015). These studies provide converging evidence by showing that performing synchronous musical activities in groups can modulate tolerance for individual pressure pain afflicted to the upper arm using a manchette for blood pressure measurement. However, the current observations are not necessarily in conflict with those previous findings. Fritz et al. (2013), for example, speculate that synchronicity between exercise movements and musical sound could be one key factor that contributed to the superior exercise performance and experience as compared to music listening to recorded music. Therefore, similar mechanisms that are believed to contribute to elevated pain-thresholds during singing and dancing in the above-cited studies may extend to workout exercise. Moreover, the findings that music listening can stimulate pleasure centers in the brain from PET-studies require participants to lay down silently and with minimal bodily movements in a scanner. At present, it seems difficult to measure and ascertain emotional brain responses to music listening during strenuous exercise.

Loud music has been identified as a potential source of hearing problems in both work and leisure environments. The size of the risk and the implications for needs of further regulation is a matter of continued and controversial debate (Morata, 2007; Zhao et al., 2010; Beach et al., 2013; Gilles et al., 2014). However, the choice of high loudness levels *per se* rests on the basic assumption that music listening induces positive effects on performance and perceived effort or strain. It is likely that this assumption must be specified in order to be of any practical use. For example, listening to loud music is generally assumed to contribute positively to fitness culture, although the research conducted to confirm this assumption is scarce and restricted to very few well-defined scenarios that do not entail the range of activities and contexts in which fitness sports happens.

Previous research on music and sports points toward a positive role of choice of tempo, which also suggests the importance of a certain coordination between auditory or audiovisual stimulation and bodily movement. Therefore, it may well be that temporal aspects such as synchronicity, tempo, and rhythm rather than sound intensity and respective loudness play a far greater role in supporting the music-aids-workout-hypothesis (Terry and Karageorghis, 2006; Fritz et al., 2013). The fitness industry already responded to such an idea by producing electronic music in specific formats to entail well-defined tempo ranges. But again, the empirical support attributing a crucial role in temporal aspects is as yet insufficient.

### Limitations

In this study, participants from a student population were invited to take part in a laboratory experiment. As is the case for a large number of psychological studies, this selection restricts the representativeness of findings to a significant degree. There are other methodological aspects that can be seen as limiting the interpretation of findings. For example, the ergometer *per se* emanates a certain type of background noise that could interfere with the music. However, increasing loudness levels also enhanced masking of the ergometer noise, but without inducing more positive effects during trials. Therefore, it seems unlikely that background noise influenced on the current findings in any systematic way. Moreover, the individual testing of the participants does not preclude a potential influence of group workout as opposed to individual workout. The presence of two experimenters and students during sessions, however, at least suggests that the presence of others *per se* might not alter the results. Finally, the music was not at an excessive loudness level and participants were exposed to the highest level (85 dB) only for few minutes according to the study protocol. Therefore, it may be that prolonged exposure to sound pressure levels above 85 dB could induce higher levels of positive affect and, consequently, a still more positive experience of the workout. However, it is obvious that the potential hearing risk outweigh the to-be-expected gains, if those exist at all.

## Conclusion

We tested the hypothesis that loud music positively influences workout at physical and subjective levels. The hypothesis was disconfirmed. Moreover, individual training status had no systematic influence on these findings. Nevertheless, there are important implications of the study. First, theories attributing a motivating role of music listening beyond distraction and entertainment during sports exercise must be revisited. Second, public policies regulating the use of music in fitness and workout contexts are advised to recommend lower levels as effective as higher levels of volume.

## Author Contributions

GK and JS conceived of and designed the research. DS and JN collected the data. DS provided a first draft. GK, JS, and AB analyzed the data and wrote the paper.

## Conflict of Interest Statement

The authors declare that the research was conducted in the absence of any commercial or financial relationships that could be construed as a potential conflict of interest.
